# Recent Progress in Flexible Pressure Sensor Arrays

**DOI:** 10.3390/nano12142495

**Published:** 2022-07-20

**Authors:** Yanhao Duan, Shixue He, Jian Wu, Benlong Su, Youshan Wang

**Affiliations:** 1 National Key Laboratory of Science and Technology on Advanced Composites in Special Environments, Harbin Institute of Technology, Harbin 150090, China; 21s130355@stu.hit.edu.cn (Y.D.); 21b908089@stu.hit.edu.cn (S.H.); subenlong@hit.edu.cn (B.S.); wangys@hit.edu.cn (Y.W.); 2 Center for Rubber Composite Materials and Structures, Harbin Institute of Technology, Weihai 264209, China

**Keywords:** flexible sensing, pressure sensors, sensor arrays

## Abstract

Flexible pressure sensors that can maintain their pressure sensing ability with arbitrary deformation play an essential role in a wide range of applications, such as aerospace, prosthetics, robotics, healthcare, human–machine interfaces, and electronic skin. Flexible pressure sensors with diverse conversion principles and structural designs have been extensively studied. At present, with the development of 5G and the Internet of Things, there is a huge demand for flexible pressure sensor arrays with high resolution and sensitivity. Herein, we present a brief description of the present flexible pressure sensor arrays with different transduction mechanisms from design to fabrication. Next, we discuss the latest progress of flexible pressure sensor arrays for applications in human–machine interfaces, healthcare, and aerospace. These arrays can monitor the spatial pressure and map the trajectory with high resolution and rapid response beyond human perception. Finally, the outlook of the future and the existing problems of pressure sensor arrays are presented.

## 1. Introduction

One of the most observable variables, pressure, is defined as the ratio of force to the area across which it is exerted, resulting in surface spatial deformation or performance variation [1]. The Earth’s gravity and physical contact contribute to the generation of pressure. Pressure sensors that can translate pressure signals into matching electrical impulses can detect pressure [2]. Flexible pressure sensors provide several benefits over rigid sensors [3,4]. Compared with conventional rigid sensors, flexible pressure sensors can withstand different deformations, such as compression, bending, tension, and even twisting, and can conform to curvilinear and even deformable surfaces [4,5,6]. Flexible pressure sensors may be produced by turning the substrate into a flexible form and employing organic and nanomaterials rather than the traditional inorganic semiconductor materials. For decades, the tremendous progress in flexible and stretchable substrates, deformable electrodes, novel durable materials, and novel processing technologies has made a huge contribution to the advancement of flexible pressure sensors. These flexible pressure sensors have distinct advantages, such as low cost, exceptional flexibility, great stretchability, and compatibility with large-area processing processes. Recently, a few novel flexible sensors have been reported to have features such as light weight, high flexibility, and fast response, which indicates an extraordinary potential for use in aerospace, prosthetics, robotics, healthcare, human–machine interfaces (HMIs), and electronic skin [7].

With the development of cyber–physical systems, the Internet of Things (IoT), and cloud computing, the concept of intelligent manufacturing has rapidly evolved [8]. Manufacturing systems in the intelligent manufacturing age may monitor physical processes and make intelligent choices through real-time communication and collaboration with humans, machines, sensors, and so on.

The rapid advancement of manufacturing systems has raised the bar for flexible pressure sensor arrays, requiring high performance, high resolution, flexibility, and low weight. With breakthroughs in artificial intelligence and computing power, intelligent systems have developed rapidly [9], necessitating a greater demand for flexible sensor arrays that can collect real-time data and transmit them to a cloud server for analysis using big data and artificial intelligence. Flexible pressure sensor arrays can visualize touch actions, track motion trajectories, and map pressure distribution in real time with high pressure sensitivity and high resolution. Therefore, there is a strong demand for flexible pressure sensor arrays with high resolution and sensitivity in order to fulfill the requirement of intelligent manufacturing, allowing issues to be solved and adaptive choices to be made on time. To broaden the detection range, improve the sensitivity, and realize the visualization of pressure imaging, enormous efforts have been applied to sensor arrays with different transduction mechanisms.

Now, the main task and problem faced by pressure sensors are those of improving their performance. High performance means that the performance parameters of the sensor are developed in continuous improvement, including high sensitivity, wide detection range, self-powered operation, high resolution, and low crosstalk. The use of new materials and the proposal of new structures have significantly improved the sensitivity and detection range of pressure sensors. High-resolution pressure sensors exceeding human skin have been achieved due to the piezo-phototronic effect and nanomaterials. To achieve the needs of self-powered operation, pressure sensors based on the piezoelectric effect and triboelectric effect have come into being. The problem of crosstalk can be addressed by rationally designing the sensor structure. However, the current achievements are still limited, and the performances of pressure sensors are expected to be further improved.

Section 2 of this review focuses on offering an introduction to transduction concepts and adaptable pressure sensor construction designs. Various transductions for pressure sensing and active materials, combined with structure designs for achieving stretchability, have enhanced the properties of flexible pressure sensors from multiple perspectives. Section 3 describes recent key advances in flexible pressure sensor arrays with various transduction concepts, from fundamental design through production. Finally, Section 4 presents the main application of flexible pressure sensor arrays, such as HMIs, healthcare, and aerospace. Furthermore, we have a projected overview of the evolution and challenges of flexible pressure sensor arrays.

## 2. Transduction Mechanisms

Flexible pressure sensors can convert external pressure stimuli into electrical signals [10]. Traditional transduction mechanisms, such as piezoresistivity, capacitance, piezoelectricity, and triboelectricity, are widely used in flexible pressure sensors. Other transduction methods are undergoing rapid development to meet new challenges and opportunities. In this section, we will briefly present four types of pressure sensors, explain the corresponding sensing mechanism and active materials, and introduce how their performance can be improved. In addition, we also summarize the main advantages and disadvantages of sensors with different transduction mechanisms.

### 2.1. Piezoresistivity

Piezoresistive pressure sensors work on the principle of converting an external pressure stimulus applied to the device into a recordable change in resistance. This effect is called the piezoresistive effect. As we know, R = ρ L/A (ρ is the material’s resistivity, L is the length, and A is the area). The L and A parameters change with the material’s deformation, which leads to a variation of the resistance value. Recently, extensive research has been carried out to obtain flexible pressure sensors with excellent performance. Traditionally, piezoresistive pressure sensors are fabricated with active materials sandwiched between two vertically aligned electrodes [11]. The performance of piezoresistive sensors can be effectively improved by selecting materials reasonably. Many conductive materials have been studied, such as metal nanowires (Ag NWs [12]), carbon-based fillers (CNTs [13], graphene [14], reduced graphene oxide [15], MXene [16]), and conductive polymers (polystyrene sulfonate (PEDOT:PSS) [17], polypyrrole [18]). Polydimethylsiloxane (PDMS) is commonly used in flexible electronic devices because it is a colorless, biocompatible, and elastic polymer [19].

At present, researchers have put forward several other methods for improving the performance of piezoresistive pressure sensors, especially by improving the sensitivity. Some researchers fabricated some microstructured flexible substrates in order to improve the performance of piezoresistive pressure sensors. The sensitivity and pressure detection range are boosted because the microstructure can cause local stress concentration, high compressibility, and easy contact area change. For example, Park et al. fabricated a conductive PDMS film with interlocked geometry of microdome arrays that were manufactured with a mixture composed of CNTs, a PDMS prepolymer, and a curing agent with a microhole-patterned silicon mold [20]. This design translates into high sensitivity to pressure (15.1 kPa^−1^~0.2 Pa minimum detection) and rapid response/relaxation times (~0.04 s). Aside from the microstructure of the microdome, some other microstructures have also been demonstrated, such as the micropyramid [21] and micropillar [22]. The performance can be also adjusted by changing the size of these microstructures [23].

Fabricating elastic, porous, and conductive materials is also an effective way to improve the sensitivity of piezoresistive pressure sensors [10]. Pan et al. produced a hollow-sphere microstructure with a polypyrrole hydrogel by using a multiphase reaction [24]. The change in resistance originates from physical contacts at discrete spots between the asperities of the conducting polymer film and the surface of electrodes. This sensor has excellent performance and can detect pressures of less than 1 Pa.

Flexible piezoresistive sensors have a number of merits, such as their simple device structure, easy read-out mechanism, low cost, and relatively simple fabrication process. However, they suffer from a large confounding temperature sensitivity and hysteresis, which limits their applications in a variety of fields.

### 2.2. Capacitance

The capacitive pressure sensor is a sensor whose capacitance changes under external pressure stimulation [25,26]. As we know, the equation for the capacitance is given by Formula (1).

(1) $$C=\varepsilon_{0}\varepsilon_{r}A/d$$

where *A* is the overlapping area, *d* is the separation gap, $\varepsilon_{0}$ is the permittivity of a vacuum, and $\varepsilon_{r}$ is the relative static permittivity of the dielectric.

During an external loading on capacitive pressure sensors, the capacitance of the sensors changes due to alterations in one or all of the three variables, that is, *ε*, *A*, and *d*. Sensitivity is one of the greatest research interests with respect to capacitive pressure sensors. The sensitivity of a parallel plate capacitor is given by Formula (2).

(2) $$s=\frac{\Delta C/C_{0}}{\Delta P}$$

S represents the sensitivity, Δ*C* is the relative change in capacitance, $C_{0}$ represents the initial capacitance when no pressure is applied, and Δ*P* is the change in the applied pressure. In order to improve the sensitivity of flexible capacitive pressure sensors, dielectric materials are engineered by adding air gaps or introducing some microstructures.

Capacitive pressure sensors are composed of a pair of conductive electrodes and a dielectric substance. Many conductive materials have been explored, such as those created by using indium tin oxide (ITO) [27], CNTs [28], metal NWs [29], and graphene [30] as electrodes and PDMS [31], polyurethane [32], and Ecoflex [33] as dielectric layers.

A high-sensitivity capacitive pressure sensor with a dielectric layer with a microstructure was fabricated by Mannsfeld et al. They used a silicon wafer with a pyramid structure as the template, and then poured the liquid PDMS into the structure in order to obtain a dielectric layer with a microstructure [34]. The stress was concentrated at the tip of the pyramid, so this sensor had better sensitivity. Then, other microstructures were explored, such as the micropillar [35] and microdome [36], which helped by providing better performance, greater compressibility, and higher sensitivity to tiny amounts of pressure.

Using a porous elastomer as the dielectric layer can effectively improve the sensitivity of capacitive pressure sensors by expelling air from the porous elastomer during compression [37]. The structure of the porous elastomer can increase the compressibility of the dielectric layer and make the dielectric layer easier to deform. In reality, the effective dielectric constant increases with compression due to the densification of pores. The effective dielectric constant ($\varepsilon_{e}$) is given in Formula (3) [38]:

(3) $$\varepsilon_{e}=\varepsilon_{\mathrm{air}}V_{\mathrm{air}}\left(P\right)+\varepsilon \;V\left(P\right)$$

where *ε*_air_ is the dielectric constant of the air, *ε* is the dielectric constant of the dielectric materials, *V*_air_ is the volume fraction of the air, and *V* is the volume fraction of the dielectric materials.

For instance, Chhetry et al. directly used an ultrasoft medical PU sponge as the dielectric layer [32]. Atalay et al. fabricated dielectrics filled with sugar and salt to create micro-porosity [39].

Flexible capacitive flexible sensors have received enormous attention because they have a simple structure, good repeatability, low loss, and temperature independence. So, they are widely applied in electronic skin, medical prosthetics, wearable devices, biometrics, touch pads, and touch screens. However, they have serious parasitic effects [40], which affect the measurement results in practical applications.

### 2.3. Piezoelectricity

Piezoelectricity is another transduction method for pressure sensing. Flexible piezoelectric pressure sensors can convert mechanical stimuli into electrical signals [41]. The piezoelectric effect refers to electric charge production due to the occurrence of electrical dipole moments from mechanical force with a non-centrosymmetric crystal structure, such as zinc oxide (ZnO), cadmium sulfide (CdS), and gallium nitride (GaN). Piezoelectric sensors comprise piezoelectric materials that are sandwiched by two parallel electrodes. Piezoelectric material deformation is caused by external force, which generates a voltage depending on the deformation. Therefore, the voltage can be used to measure the force applied to the sensors. Wang and Song first fabricated a piezoelectric nanogenerator (PENG) based on zinc oxide nanowires (ZnO NWs) in 2006 [42]. Piezoelectric sensors have various applications. Some piezoelectric materials have been used in flexible sensors—for example, inorganic nanomaterials, such as metal materials, nanowires [43], and nano-thin films [44], and organic nanomaterials, such as polyvinylidene fluoride (PVDF) [45] and poly(vinylidenefluoride-co-trifluoroethylene) P(VDF-TrFE) [46]. These piezoelectric materials can not only serve as sensing materials, but also generate energy from the environment.

Flexible piezoelectric sensors have high sensitivity, low power consumption, and excellent dynamic response, making them promising candidates for self-powered sensors. In contrast, piezoelectric sensors are unsuitable for static measurements.

### 2.4. Triboelectricity

Triboelectric nanogenerators (TENGs) have recently gained much attention due to their exceptional performance in energy harvesting and signal induction. Furthermore, TENGs have recently sparked much curiosity, ever since Wang et al. identified the first one in 2012 [47]. TENGs have an excellent application in self-powered sensors and power supplies. The principle of TENGs is based on the coupling effect of electrification and electrostatic induction. Electron transition between the atoms/molecules happens with a strong electron cloud overlap (or wave function overlap) between the two atoms/molecules in the repulsive region owing to the reduced interatomic potential barrier [48]. TENGs consist of positive and negative triboelectric layers, electrodes, and auxiliary structures, including substrates, spacers, and wires. When two materials make contact, opposite static charges form on their surfaces, and electrons travel across the external circuit owing to potential differences. There are four different working modes of TENGs: the vertical contact-separation mode, lateral sliding mode, the single-electrode mode, and the freestanding mode.

Numerous materials have been investigated for the fabrication of triboelectric pressure sensors, such as PDMS [49], PU [50], hydrogels [51], indium tin oxide (ITO) [52], and fabrics [53]. The friction surfaces have been micro-nano structured to increase the performance of triboelectric sensors [54,55,56,57]. The triboelectric effect is significantly boosted because the contact area between the two materials is enhanced. For example, a triboelectric sensor was made using a modified PDMS with hierarchical polymeric architectures of a nanoporous and interlocked microridge structure [58]. By utilizing this, the sensor exhibited power density (46.7 μW/cm^2^), pressure (0.55 V/kPa), and bending (~0.1 V/°) sensitivities.

Flexible triboelectric sensors have the merits of high sensitivity, low energy cost, simple manufacturing process, and the ability to be self-powered. However, triboelectric sensors can only sense dynamic forces.

## 3. High-Performance Flexible Pressure Sensor Arrays: Design and Fabrication

### 3.1. Piezoresistive Pressure Sensor Arrays

In order to detect pressure distributions for applications in electronic skin, robotics, HMIs, and the aerospace field, it is necessary to fabricate pressure sensor arrays with a high resolution, high sensitivity, fast response, and low cost. Compared to sensors with other conversion mechanisms, piezoresistive sensor arrays require a less complex data acquisition system and are less sensitive to electromagnetic noise [59]. This part will discuss the recent progress in flexible piezoresistive pressure sensor arrays.

**Figure 1:**
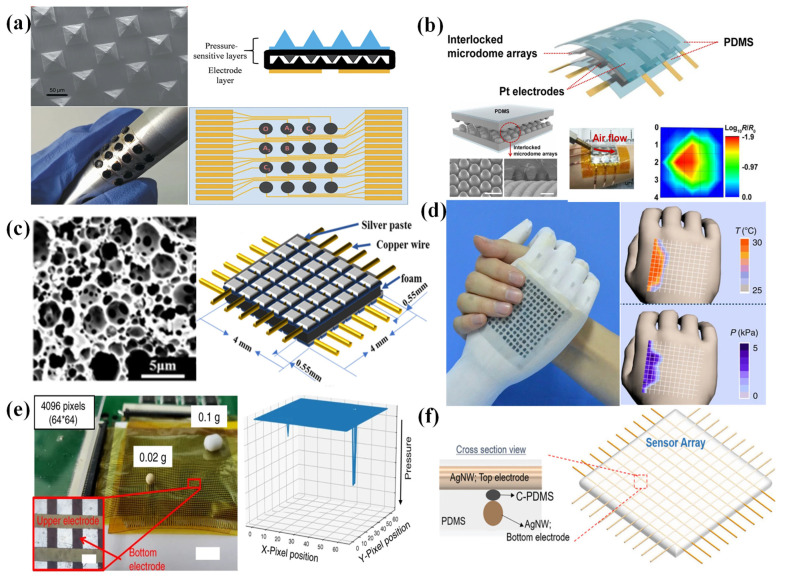
(**a**) A PDMS/MWCNT-based tactile sensor array with coplanar electrodes for crosstalk suppression. Reproduced with permission from Ref. [60]. Copyright 2016, Luxian Wang et al. (**b**) A tactile-direction-sensitive and stretchable electronic skin based on human-skin-inspired interlocked microstructures. Reproduced with permission from Ref. [61]. Copyright 2014, American Chemical Society. (**c**) A sensor array based on a highly sensitive, broad-range, hierarchically structured reduced graphene oxide/PolyHIPE foam. Reproduced with permission from Ref. [63]. Copyright 2019, American Chemical Society. (**d**) A flexible and self-powered dual-parameter temperature–pressure sensor array using microstructure-frame-supported organic thermoelectric materials. Reproduced with permission from Ref. [64]. Copyright 2015, Fengjiao Zhang et al. (**e**) A quantum-effect-based flexible and transparent pressure sensor array with ultrahigh sensitivity and sensing density. Reproduced with permission from Ref. [65]. Copyright 2020, Lan Shi et al. (**f**) A behavior-learned cross-reactive sensor matrix for intelligent skin perception. Reproduced with permission from Ref. [66]. Copyright 2020, WILEY-VCH.

Wang et al. fabricated a piezoresistive pressure sensor array whose piezoresistive layers were designed with a micropyramid structure to improve the sensitivity [60]. As the sensor density of the sensing array increased, signal interference (crosstalk) was generated due to the reduced analog signal strength, in addition to an increase in the number of interconnects and a reduction in the spacing between interconnects [7]. The problem of crosstalk could be solved by combining the isolated sensing elements in the coplanar electrode layers. The fabrication process (Figure 1a), including the fabrication of a pressure-sensitive layer with micropyramids and a coplanar Au electrode layer, was simple. However, this structure requires a large number of electrode wires—for instance, an n × m sensor array needs n × m wires. There are many problems in the fabrication and miniaturization of sensor arrays.

Due to the weakness mentioned above, a new strategy based on the cross-type electrode configuration was introduced. Only n + m wires would be needed for an n × m sensor array. For example, inspired by the interlocked microstructures of human skin, a sensor array with interlocked piezoresistive microdome arrays was proposed by Park et al. [61]. Because of the unique geometry of interlocked microdome arrays, this device could detect not only normal pressure, but also shear, tensile, bending, and torsional forces. A 3 × 3 sensor array sandwiched between cross-arrays of platinum electrodes was fabricated (Figure 1b) with a sputter-coating system, and it could provide a spatially resolved mapping of the touch positions. Similarly, a sensor array with the structure of a multilayer interlocked microdome geometry was proposed, and this device could sense linearly over a broad pressure range (0.0013–353 kPa) with a fast response time (20 ms) [62]. To fabricate a sensor array with 4 × 8 pixels, the sputter-coating system coated platinum electrodes on substrates, and the films with multilayer interlocked microdomes were sandwiched by the patterned electrodes. By using a cross-type electrode configuration, a new piezoresistive sensor fabricated from a foam with a hierarchical pore size distribution was also proposed by Yang et al. [63]. The composite foams were synthesized via high-internal-phase emulsion polymerization (PolyHIPE) in the presence of reduced graphene oxide (rGO). It was capable of high sensitivity over a pressure range spanning from 0.6 Pa to 200 kPa because of the structural design of the hierarchical pores. To fabricate a 6 × 6 pixel array, silver paste electrodes were screen-printed onto the surfaces of the rGO@PolyHIPEs and connected to copper wires (Figure 1c). Zhang et al. fabricated a dual-parameter temperature–pressure sensor with an accurate temperature resolution of 0.1 K and a high pressure-sensing sensitivity of up to 28.9 kPa^−1^ [64]. The sensors could obtain temperature and pressure-sensing properties because the active layer consisted of organic thermoelectric materials deposited on a deformable microstructure frame. The temperature difference could be detected via the thermoelectric effect, and the pressure could be observed through a change in the resistance of the active layer. The dual-parameter sensor array was constructed by using a simple stamp-printing method, and it could obtain spatially resolved images with both temperature and pressure (Figure 1d). A semitransparent fabric was used as the microstructured frame. The high-resolution sensor array with remarkable flexibility was fabricated by inkjet-printing PEDOT:PSS on the semitransparent fabric and using a polyethylene terephthalate film (PET) with a patterned Ti/Au array as electrodes.

The sensing pixel of a piezoresistive sensor array is associated with the size of the surface microstructure. So, one pixel of these sensor arrays with a surface microstructure may be interfered with by adjacent pixels, which limits the sensing density. So, a thin-film piezoresistive pressure sensor based on the Fowler–Nordheim tunnelling effect was fabricated by spin-coating extremely low urchin-like hollow carbon spheres dispersed in polydimethylsiloxane [65]. This pressure sensor had an ultrahigh sensitivity of 260.3 kPa^−1^ at 1 Pa, high transparency, and temperature noninterference. Through magnetron sputtering to obtain electrodes, a 64 × 64 sensing array was fabricated with the ability to distinguish the position, different weight, and subtle differences in shape (Figure 1e).

In order to further improve the resolution of the entire sensor array, some studies used conductive threads as electrodes. Sundaram et al. integrated a sensor array on a knitted glove to explore the mechanics of how humans grasp objects and the typical tactile patterns that emerge while grasping objects. A force-sensitive film connected by a network of conductive threads was attached to a custom-knit glove designed not to interfere with hand movements. There was a change in the film resistance between the row and column electrodes when the glove touched an object [67]. As shown in Figure 1f, by using highly stretchable cross-aligned silver nanowire for the electrodes and carbon black)-dispersed poly(dimethylsiloxane) as a piezoresistive layer, a highly stretchable cross-reactive sensor array with high sensitivity and fast responses was demonstrated [66]. In addition to pressure, this device could also detect strain, flexion, and temperature. Similarly to using conductive wires as electrodes, some studies used composite fibers as both sensing elements and conductive electrodes [68]. A piezoresistive sensor array based on three-layer core–shell Ag/AgCl/PEDOT: PSS composite fibers was demonstrated by Wang et al. [69]. This sensor had an excellent performance with a response time of 32 ms and a sensitivity of 5.12 kPa^−1^. The composite fibers were woven into an electronic fabric, and a resistance analyzer was connected to the fabric to compose the sensor array.

### 3.2. Capacitive Pressure Sensor Arrays

Compared with pressure sensors with other transduction mechanisms, capacitive pressure sensors have gained extensive attention because they have a simple structure, high sensitivity, temperature independence, relatively low power consumption, and good dynamic responses. This part will discuss the recent progress of flexible capacitive pressure sensor arrays.

**Figure 2:**
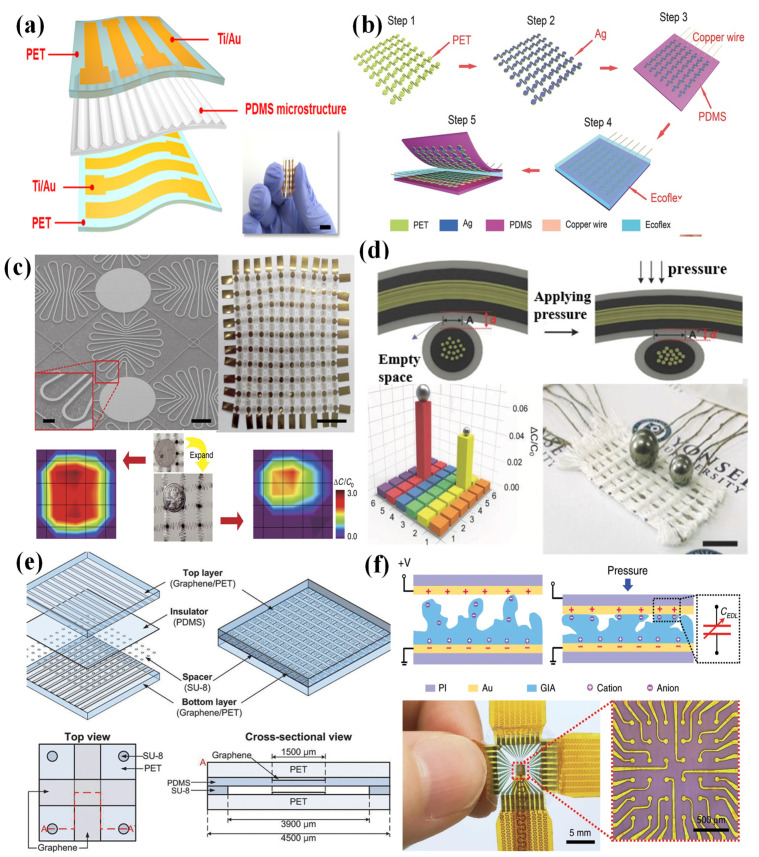
(**a**) A tunable, ultrasensitive, and flexible pressure sensor array based on wrinkled microstructures. Reproduced with permission from Ref. [71]. Copyright 2019, American Chemical Society. (**b**) A flexible, stretchable, and wearable multifunctional sensor array for static and dynamic strain mapping. Reproduced with permission from Ref. [72]. Copyright 2015, WILEY-VCH. (**c**) A skin-inspired highly stretchable and conformable matrix network for multifunctional sensing. Reproduced with permission from Ref. [73]. Copyright 2018, Qilin Hua et al. (**d**) A conductive fiber-based ultrasensitive textile pressure sensor. Reproduced with permission from Ref. [74]. Copyright 2015, WILEY-VCH. (**e**) A flexible, transparent, sensitive, and crosstalk-free capacitive tactile sensor array based on graphene electrodes and air as a dielectric. Reproduced with permission from Ref. [75]. Copyright 2017, WILEY-VCH. (**f**) A graded intrafillable-architecture-based iontronic pressure sensor array with ultra-broad-range high sensitivity. Reproduced with permission from Ref. [76]. Copyright 2020, Ningning Bai et al.

By using a cross-type electrode configuration, some capacitive sensor arrays with excellent properties have been presented. A breathable and screen-printed pressure sensor array based on nanofiber membranes was demonstrated by Yang et al. [70]. Benefiting from the high porosity of nanofiber membranes, this sensor had a superior sensitivity of 4.2 kPa^−1^, a fast response time (<26 ms), and an ultralow detection limit (1.6 Pa). Using PVDF nanofiber membranes as substrates, silver nanowires as electrodes, and thermoplastic polyurethane nanofiber membranes as the dielectric layer, the pressure sensor array was obtained with screen-printing and ultrasonic bonding techniques. Then, a tunable pressure sensor with a microstructure of wrinkles was demonstrated by Zeng et al. [71]. The sensitivity and working range of this sensor could be tuned by the method of changing the aspect ratio and amplitude of the microstructure. Then, by adding hollow structures in the wrinkles, the sensitivity of this sensor could reach 14.268 kPa^−1^. The electrode was obtained by depositing the Ti and Au on the PET substrates, and then the microstructured PDMS with a wrinkle pattern was generated by using a micropatterned mold. A 4 × 4 sensor array was acquired through the orthogonal placement of two PET substrates with patterned Ti/Au electrodes (Figure 2a).

In order to improve the stretchability and resolution of sensor arrays, some studies changed the electrodes of capacitive sensor arrays. Zhao et al. fabricated a capacitive pressure sensor array through a simple fabrication procedure [72]. Ag thin films were sputtered on serpentine-cut PET sheets as electrodes, and then electrodes were pasted on the PDMS substrate to pattern a 7 × 7 sensor array. Finally, Ag/PET/PDMS film was placed orthogonally to another identical Ag/PET/PDMS film—face to face—forming a sandwich structure with Ecoflex in the middle (Figure 2b). Inspired by skin, a highly stretchable and conformable matrix network was proposed [73]. It could be expanded to several orders of magnitude compared to the original area because of the structure of the meandering interconnect. The pressure sensor array was fabricated on a side of a PI substrate, which was composed of an Ecoflex dielectric layer sandwiched between Ag thin film electrode layers. This sensor array could be used to identify the position of a pressure load and to estimate the size of the loading object before and after a 300% expansion of the network (Figure 2c).

To further increase the resolution, a capacitive pressure sensor array based on the conductive fibers was demonstrated by Lee et al. [74]. A 6 × 6 array was fabricated by using PDMS-coated conductive fibers to weave the sensor array by using a weaving technique (Figure 2d). This sensor array could detect the spatial distribution of external pressure because the capacitance of the crossing point of the conductive fibers changed with increased pressure.

Crosstalk significantly diminishes the measurement accuracy. To reduce the problem of crosstalk, Pyo et al. proposed a flexible, fully transparent, and highly sensitive capacitive sensor array with the structure of two sets of facing graphene electrodes separated by spacers [75]. Figure 2e shows the design of the transparent capacitive sensor array, and there are two graphene-patterned PET layers, a PDMS insulator, and SU-8 spacers. Because of the air gap between the graphene electrodes, the sensor had a high sensitivity of 6.55% kPa^−1^ and a fast response/relaxation time of 70 ms. Using spacers to isolate each cell allowed this sensor array to measure the spatial distribution without crosstalk.

When sensors are scaled down to the microscale, the signal of capacitive pressure sensors is often susceptible to noise. In order to solve this problem, Bai et al. fabricated an iontronic sensor with an intrafillable structure [76]. Because of the high intrafillability of the structure, this sensor exhibited high sensitivity ($S_{min}$ > 220 kPa^−1^) over a broad pressure regime (0.08 Pa–360 kPa). Patterned Au electrodes were obtained by usingthe method of photoresist mask. A sensor array of 6 × 6 pixels, with each sensing pixel being a circular sensor of 60 μm, was fabricated, and it allowed for high-resolution pressure sensing with a high signal-to-noise ratio (Figure 2f). However, compared with the cross-type electrode configuration, this method requires more addressing lines, thus presenting further challenges in achieving fast mapping.

### 3.3. Piezoelectric Pressure Sensor Arrays

With the serious problems of the energy crisis and global warming, renewable clean energy is a popular topic because of its sustainability and environmental friendliness, so self-powered sensors have attracted significant attention. A PENG can convert mechanical energy from the surroundings into electrical energy, so it is a promising candidate for self-powered sensors. Researchers have been working to develop piezoelectric sensor arrays with better performance in recent decades. This part will discuss the recent progress of flexible piezoelectric pressure sensor arrays.

**Figure 3:**
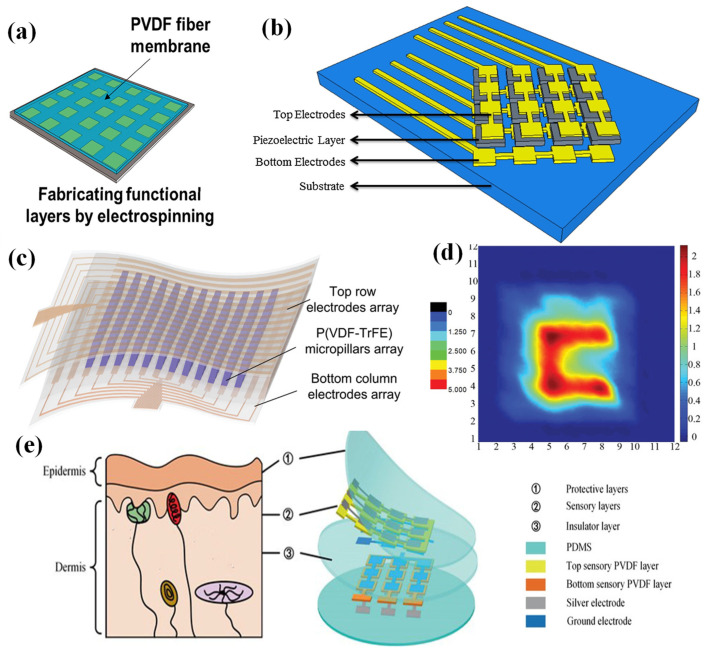
(**a**) A bionic single-electrode electronic skin unit based on a piezoelectric nanogenerator. Reproduced with permission from Ref. [77]. Copyright 2018, American Chemical Society. (**b**) A screen printing of a flexible piezoelectric-based device on polyethylene terephthalate and paper for touch- and force-sensing applications. Reproduced with permission from Ref. [79]. Copyright 2017, Elsevier. (**c**) A scalable imprinting of a flexible multiplexed sensor array with distributed piezoelectricity-enhanced micropillars for dynamic tactile sensing. Reproduced with permission from Ref. [80]. Copyright 2020, WILEY-VCH. (**d**) A flexible sensor array used for touch applications and mapping of force distribution. Reproduced with permission from Ref. [80]. Copyright 2020, WILEY-VCH. (**e**) A skin-inspired piezoelectric tactile sensor array with crosstalk-free row + column electrodes for spatiotemporally distinguishing diverse stimuli. Reproduced with permission from Ref. [81]. Copyright 2021, Weikang Lin et al.

Wang et al. fabricated a single-electrode piezoelectric pressure sensor array by using electrospun PVDF nanofibers [77]. The electrode array was fabricated on an insulating substrate material by magnetron sputtering, and then the functional layer of PVDF was formed on the electrode layer by electrospinning (Figure 3a). This sensor array could sense not only pressure signals, but also temperature signals. The single-electrode sensor array had good ductility, but this structure needed more addressing lines, which hindered the fabrication of sensor arrays with more pixels.

The most common structure of piezoelectric sensor arrays is designed as a cross-type configuration of electrodes. In 2013, a transparent piezoelectric sensor array was fabricated with a screen-printing routine by Zirkl et al. [78]. A ferroelectric copolymer (PVDF: TrFE) was fully printed on a plastic foil, and then silver conductive lines were printed to connect the electrodes for signal readout. This device could detect changes in temperature and pressure. Then, Emamian et al. fabricated a piezoelectric sensor array with sensitivities of 1.2 and 0.3 V/N, respectively, on flexible PET and paper substrates through the screen-printing technique [79]. As shown in Figure 3b, a screen-printed PVDF was sandwiched between the printed top and bottom Ag electrode metallization layers. This device could be used for both touch- and force-based applications. Some microstructures have been investigated for the improvement of the performance of piezoelectric sensor arrays. For instance, Chen et al. fabricated a sensor array based on piezoelectricity-enhanced vertically aligned P(VDF-TrFE) micropillars [80]. Each sensor pixel had uniform output generation, robust output stability, and scalable fabrication ability. Compared to planar films, the sensor showed an enhanced sensitivity of 228.2 mV N^−1^ and a highly linear load response. Piezoelectric micropillar array films with a diameter of 5 µm and a height of 50 µm were fabricated with a nanoimprinting technology, and then sandwiched between a pair of cross-electrode arrays to construct a 12 × 12 sensor array (Figure 3c). The electrode arrays were fabricated using lithography, sputtering, and a lift-off process, and the P(VDF-TrFE) film was imprinted on a PDMS mold with microcave arrays to fabricate the piezoelectric micropillar arrays. Finally, the electrode arrays and the piezoelectric micropillar arrays were assembled to form the sensor array. This sensor array could track a dynamic force and image the force distribution in real time with a portable signal processing circuit (Figure 3d).

In order to overcome the issue of crosstalk, a new strategy for piezoelectric sensor arrays was introduced. Inspired by human skin, a piezoelectric sensor array with zero crosstalk was proposed by Lin et al. [81]. As shown in Figure 3e, this device had a multilayer architecture that consists of two PDMS films serving as the protective layers, two PVDF films as the sensory layers, and one PDMS film as the insulative layer. Using the PDMS as an insulative layer could prevent crosstalk between the two PVDF layers. This sensor array had a high sensitivity of 7.7 mV kPa^−1^, a fast response of 15 ms, long-term durability, and zero crosstalk.

Moreover, to achieve the goal of high-resolution flexible and visual pressure sensing, flexible pressure sensor arrays based on the piezo-phototronic effect, which is defined as the coupling effect of piezoelectricity and photoexcitation properties, have been proposed. A sensor array based on the piezo-phototronic effect can convert mechanical stress into visible light emission with a high resolution.

**Figure 4:**
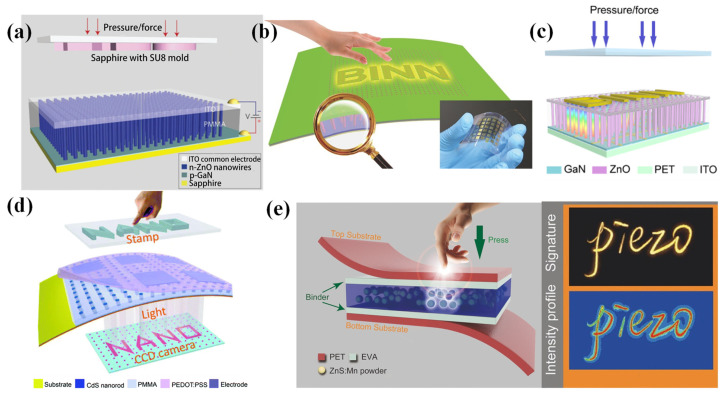
(**a**) High-resolution electroluminescent imaging of pressure distribution using a piezoelectric nanowire LED array. Reproduced with permission from Ref. [82]. Copyright 2013, Nature Publishing Group. (**b**) A flexible and controllable piezo-phototronic pressure-mapping sensor matrix with a ZnO NW/p-Polymer LED array. Reproduced with permission from Ref. [83]. Copyright 2015, WILEY-VCH. (**c**) Achieving high-resolution pressure mapping via a flexible GaN/ZnO nanowire LED array with the piezo-phototronic effect. Reproduced with permission from Ref. [84]. Copyright 2019, Elsevier. (**d**) A CdS nanorod/organic hybrid LED array and the piezo-phototronic effect of the device for pressure mapping. Reproduced with permission from Ref. [85]. Copyright 2016, Royal Society of Chemistry. (**e**) A dynamic pressure mapping of personalized handwriting by a flexible sensor array based on the mechanoluminescence process. Reproduced with permission from Ref. [86]. Copyright 2015, WILEY-VCH.

In 2013, Pan et al. developed a pressure sensor array with a high resolution of 2.7 mm based on the piezo-phototronic effect by using a piezoelectric nanowire light-emitting diode (LED) [82]. Every pixel was composed of an n-ZnO nanowire/p-GaN light-emitting diode, and n-ZnO nanowires were grown on a p-GaN thin film substrate on sapphire (Figure 4a). The pressure could control the emission intensity, and the pressure image could be obtained by the signals of all of the pixels of electroluminescence. Afterwards, as shown in Figure 4b, a flexible ITO/PET substrate was used instead of a conventional rigid GaN/sapphire substrate [83]. So, a flexible pressure sensor array composed of a PEDOT: PSS layer and a patterned n-type ZnO nanowire array was fabricated. This device could map spatial pressure distributions with a spatial resolution of 7 µm, and the range of pressure detected could be changed by controlling the growth conditions of the ZnO nanowire array. The piezopotential created by the external pressure tuned the energy band on the ZnO side and dramatically increased the recombination rate of charge carriers in the ZnO nanowires, which increased the light-emission intensity. As shown in Figure 4c, a sensor array based on a p-GaN film/n-ZnO nanowire LED, with the merits of flexibility, a high resolution of 2.6 µm, a fast response of 180 ms, stability, and a light weight, was also investigated [84]. The sensor array could be fabricated through the laser lift-off process and the hydrothermal growth of ZnO nanowires on a flexible GaN film.

CdS has attracted much attention and is widely used in light-emitting diodes because of its nonlinear properties and piezo-electronic effect. Bao et al. fabricated a piezo-phototronic effect pressure sensor array on a flexible Au/Cr/Kapton substrate using a flexible CdS nanorod/PEDOT:PSS LED array (Figure 4d) [85]. This pressure sensor array could map the applied pressure with a high spatial resolution of 1.5 μm. Because of the mechanoluminescence of ZnS:Mn particles (ZMPs), ZMPs can be also used to fabricate pressure sensor arrays. ZMPs were used to fabricate a pressure sensor array with a fast response time of less than 10 ms and a high spatial resolution of 100 µm (Figure 4e) [86]. This device could be used as a more secure electronic signature collector to record handwritten signatures and the signing behavior/habits of signers.

### 3.4. Triboelectric Pressure Sensor Arrays

Traditional stiff sensors are limited by external power supplies provided by rigid batteries with a heavy weight and colossal occupation of space. With the development of biomedical therapy, wearable devices, and medical healthcare, it is essential to develop self-powered sensors that can function sustainably and continuously without an external power source [87]. TENGs have the ability to be self-powered because they can gather mechanical energy based on the triboelectric effect. Recently, endeavors have been devoted to improving the wide detection range, high spatial resolution, and visualization performance of triboelectric sensors. This part will discuss the recent progress in flexible triboelectric pressure sensor arrays.

**Figure 5:**
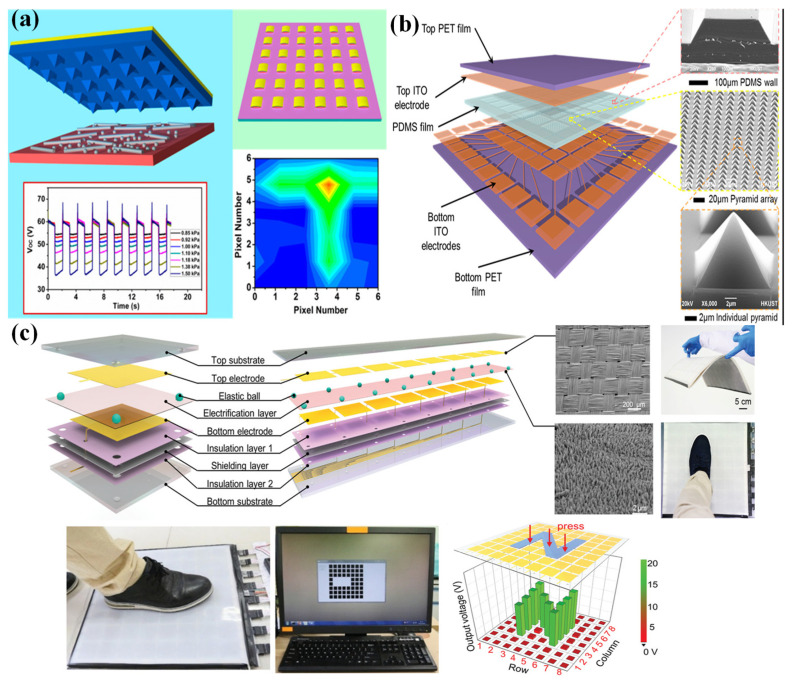
(**a**) A triboelectric sensor array for self-powered static and dynamic pressure detection and tactile imaging. Reproduced with permission from Ref. [88]. Copyright 2013, American Chemical Society. (**b**) An integrated flexible, waterproof, transparent, and self-powered tactile sensing panel. Reproduced with permission from Ref. [93]. Copyright 2016, American Chemical Society. (**c**) A large-area integrated triboelectric sensor array for wireless static and dynamic pressure detection and mapping. Reproduced with permission from Ref. [94]. Copyright 2019, John Wiley and Sons.

Initially, the first triboelectric pressure sensor array based on the vertical contact-separation mode was fabricated by Lin et al. in 2013 [88]. By integrating an active triboelectric sensor unit into the same electrode, the pressure sensor array could monitor and map the local pressure distribution applied to the device (Figure 5a). The response of this device to local pressure could be measured through a multichannel measurement system. However, each pixel of this sensor was packaged independently.

However, this integration method based on the contact-separation mode is not suited to the large-scale fabrication of high-resolution sensor arrays because of the cumbersome fabrication process for each pixel. The development of triboelectric sensor arrays based on single-electrode mode provides an excellent way to solve this problem [89].

**Figure 6:**
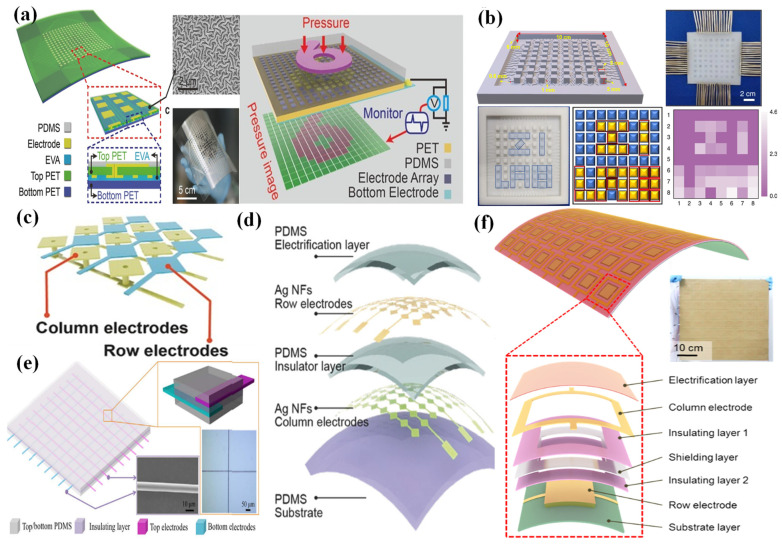
(**a**) A self-powered, high-resolution, and pressure-sensitive triboelectric sensor array for real-time tactile mapping. Reproduced with permission from Ref. [91]. Copyright 2016, WILEY-VCH. (**b**) A metal-electrode-free, fully integrated, soft triboelectric sensor array for self-powered tactile sensing. Reproduced with permission from Ref. [92]. Copyright 2020, Lingyun Wang et al. (**c**) Electrode structural design of the cross type. Reproduced with permission from Ref. [91]. Copyright 2016, WILEY-VCH. (**d**) A highly stretchable, transparent, and self-powered triboelectric tactile sensor with metallized nanofibers. Reproduced with permission from Ref. [95]. Copyright 2017, WILEY-VCH. (**e**) A self-powered sensor array for high-resolution pressure sensing. Reproduced with permission from Ref. [97]. (**f**) Triboelectrification-enabled touch sensing for self-powered position mapping and dynamic tracking with a flexible and area-scalable sensor array. Reproduced with permission from Ref. [98]. Copyright 2017, Elsevier.

The first single-electrode-mode triboelectric pressure sensor array was proposed by Yang et al. in 2013 [90]. ITO electrodes were separated to fabricate the array configuration and were then entirely covered by a PDMS film as a touch-sensitive unit. The pressure sensor array could track the location and pressure of human touch. Various microfabrications and sophisticated designs in the structure could be used to reduce the size of the electrode patch. Then, Wang et al. explored a pressure-sensitive triboelectric sensor array with a resolution of 5 dpi and a high sensitivity of 0.06 kPa^−1^, which could detect a single-point and multi-point pressure distribution in real time [91]. A piece of PET film was used as a flexible substrate, and Ag electrodes were deposited on the substrate. The top electrodes served as charge-sensing elements, and the electrodes on the bottom side connected to external measuring equipment. Another bottom PET film coated with ethylene–vinyl acetate copolymer was utilized as an encapsulation layer of the electrodes in the circuit configuration. A PDMS film was spin-coated onto the top, serving as an electrification layer that could generate triboelectric charges when touched. Figure 6a provides a schematic of this flexible self-powered triboelectric sensor array. Similarly to this structure, a polymer-integrated triboelectric sensor array was proposed by Wang et al. [92]. A metal-electrode-free sensor array with an 8-by-8 sensor could be fabricated by using a low-cost approach. Ecoflex 0–30 or 0–50 was poured into a template with a designed pattern composed of 8-by-8 sensor units and serpentine electrode lines, and then the cavities were filled with a mixture of PVA/PEI gel (Figure 6b). This device had a remarkable sensitivity of 0.063 V kPa^−1^ with a linear range from 5 to 50 kPa.

However, this structure needed several addressing lines, which caused problems when increasing the pixels and enlarging the scale of sensor arrays. So, a new method based on the cross-type configuration was developed by Wang et al. to reduce the addressing lines to m + n and simplify the structure. The corresponding schematic design of the 16 × 16 pixelated triboelectric sensor array is shown in Figure 6; the ratio of an electrode’s area in each pixel could be enhanced with a rhombic array configuration [91]. Using a multi-switch scanning method, voltage signals from both the corresponding row and column electrodes could be measured. So, this device could achieve the goal of the pressure-mapping process. After that, many triboelectric array sensing systems with similar structures appeared. For example, by using patterned Ag nanofiber as electrodes, a self-powered, highly stretchable, and transparent sensor array was proposed by Wang et al. [95]. The Ag nanofibers could be fabricated by electrospinning and with a photolithography technique, and column and row electrodes were employed with a crossbar-type configuration (Figure 6d). A cross-locating technology was utilized to realize rapid pressure mapping. A triboelectric sensor array with a simple structure based on cascaded row + column electrodes embedded in porous silicone rubber with a low modulus was investigated [96]. A textile coated with copper was shaped as rows + columns by using laser cutting and was embedded between porous silicone rubber and silicone substrate. This device had the ability to cover large areas with increased spatial resolution in both single-pixel and multipixel sensing by using the row-scanning method.

To improve the resolution of sensor arrays, by using carbon fibers as electrodes, an ultrahigh-resolution—as high as 127 × 127 dpi—pressure sensor array that was capable of mapping the 2D tactile trajectory of a tip was demonstrated by Ma et al. [97]. Two groups of carbon fibers were equidistantly distributed along the vertical direction, and a thin PDMS film was used as an insulating layer (Figure 6e). Although the sensor array had an ultrahigh resolution, the sensitivity was low, and the triboelectric effect was poor.

By using a proper shielding design, Zhu et al. proposed a sensor array with extremely low crosstalk between adjacent pixels [98]. As shown in Figure 6f, a nickel-deposited conductive fabric was used to shield the two electrode layers. The electrostatic induction between two electrode layers could be eliminated because of the shielding layer, so the crosstalk could be largely eliminated. The size of the shielding layer was smaller than that of the column electrode, but larger than that of the row electrode. An output voltage of up to 25 V could be achieved on both the row and column electrode lines.

Although there have been plentiful efforts aimed at improving the performance of triboelectric sensor arrays, the wider detection range, higher resolution, and visualization still need to be improved because of the need for practical application. Consequently, in order to improve the performance of triboelectric pressure sensor arrays, some researchers have come up with combinations of the triboelectric effect with other mechanisms.

**Figure 7:**
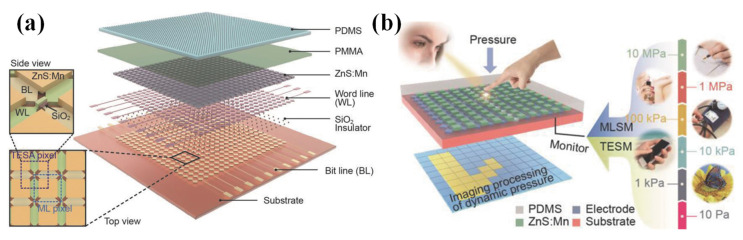
(**a**) A full-dynamic-range pressure sensor matrix based on dual-mode optical and electrical sensing. Reproduced with permission from Ref. [99]. Copyright 2017, WILEY-VCH. (**b**) Diagram of pressure regimes and the relevant applications in our daily lives. Reproduced with permission from Ref. [99]. Copyright 2017, WILEY-VCH.

Aimed at improving the detection range, a full-dynamic-range pressure sensor array was fabricated by integrating the two modes of the triboelectric effect and mechanoluminescent mechanism, which simultaneously exhibited a high sensitivity and resolution [99]. This device could achieve a resolution of 100 dpi and an excellent sensitivity of 6 MPa^−1^ from 0.6 to 200 kPa and 0.037 MPa^−1^ from 650 kPa to 30 MPa. The device had two parts: a triboelectric sensor array and a mechanoluminescent sensor array. The triboelectric sensor array was used to detect low pressure regimes, and the mechanoluminescent sensor array was used to detect high pressure. The basic structure of this device is shown in Figure 7a. An electrode with a rhombic array configuration was used to enhance the mapping rate and effective electrode area ratio in each pixel. Then, a ZnS: Mn powder and photoresistor on the top of the electrodes were designed for a mechanoluminescent sensor array. Finally, a PDMS with a modified microstructure acted as the triboelectric film. This device could mainly cover the entire pressure range in our daily lives (Figure 7b), and it could also achieve dynamic pressure mapping.

In addition to combining the triboelectric effect and mechanoluminescent mechanism, a tribotronic transistor [100,101,102,103] was proposed by using the electrostatic potential of the triboelectric effect as a gate voltage to control charge carriers’ transport. Similarly, a new combination of the triboelectric effect and electroluminescence was also proposed [104,105,106,107]. It could visualize dynamic motions with a triboelectrification-induced electroluminescence sensor.

## 4. Application of Flexible Pressure Sensor Arrays

Here, applications of flexible pressure sensor arrays in the HMI, healthcare, and aerospace fields are described systematically.

### 4.1. Human–Machine Interface

A Human–machine interface (HMI) is a new technology that can transfer information between humans and electronic devices. Recently, advancements in the IoT and artificial intelligence have put forward new requirements for HMIs, such as flexibility, portability, etc. An HMI can help achieve the goal of harmonious coexistence and efficient collaboration between humans and the digitalized world [108,109]. Pressure sensor arrays are a significant part of an HMI, and recent advances in HMIs have been achieved by using flexible pressure sensor arrays.

**Figure 8:**
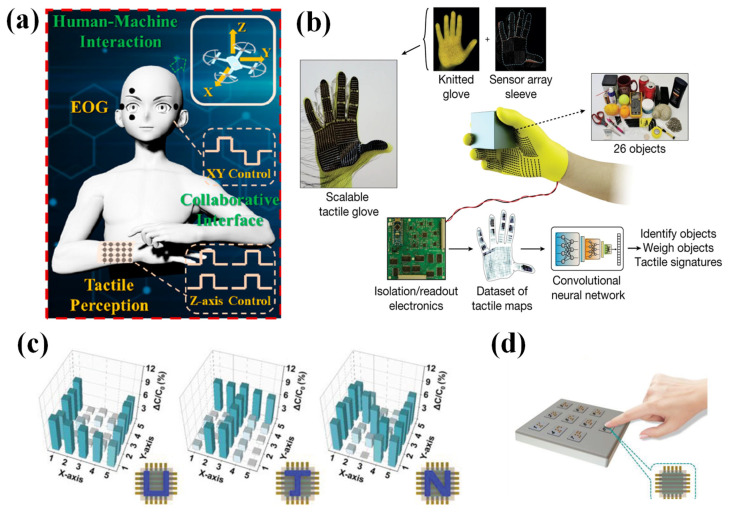
(**a**) Electrooculography and tactile perception in a collaborative interface for 3D human–machine interaction. Reproduced with permission from Ref. [110]. Copyright 2022, American Chemical Society. (**b**) A scalable tactile glove consisting of a sensor array with 548 elements covering the entire hand. Reproduced with permission from Ref. [67]. Copyright 2019, Subramanian Sundaram et al. (**c**) Spatial pressure distribution capability test of a 5 × 5 sensor array using plastic boards that are shaped like the letters “U”, “J”, and “N”. Reproduced with permission from Ref. [111]. Copyright 2019, WILEY-VCH. (**d**) An image of a numeric keypad with braille. Reproduced with permission from Ref. [111]. Copyright 2019, WILEY-VCH.

A vital application of flexible pressure sensor arrays in HMIs is spatial pressure distribution monitoring and real-time trajectory mapping. For example, Xu et al. proposed a 3D human–machine interaction by combining electrooculography (EOG) and a 4 × 4 capacitive pressure sensor array [110]. The signals of the EOG were used for convenient contactless 2D (XY-axis) interaction, and the signals of the pressure sensor array were used for complex 2D movement control and Z-axis control in the 3D interaction (Figure 8a).

Motivated by the regular use of human hands for daily interaction, smart gloves are predicted to replace conventional HMIs, such as keyboards, joysticks, etc. [112]. Smart gloves are also an important applications of HMIs. For instance, Sundaram et al. demonstrated a tactile glove that was assembled with piezoresistive pressure sensor arrays on a knitted glove [67]. A deep convolutional neural network and a scalable tactile glove were combined to reveal the mechanics of how humans grasp objects. The tactile glove could record tactile videos and detect normal forces (Figure 8b); more importantly, the tactile glove’s cost was low.

Niu et al. fabricated a 5 × 5 capacitive pressure sensor array with a spatial-pressure differentiation ability [111]. The sensor array could distinguish objects with the shapes of “U”, “J”, and “N” (Figure 8c). A numeric keypad in braille was proposed by combining it with the capacitive pressure sensor array (Figure 8d). Moreover, the braille could be decoded through the braille numeric keypad when an artificial fingertip touched the braille. Combined with a TENG)and a field-effect synaptic transistor, a 28 × 28 sensor array with a high sensitivity of 0.192 kPa^−1^ in the range of 1 to 5 kPa and 0.007 kPa^−1^ in the range of 5 to 20 kPa was presented [103]. This device could realize handwritten images that were visualized in real time in addition to subsequent real-time neuromorphic computing.

### 4.2. Healthcare

Significant advances in wearable technologies are revolutionizing our lives by integrating flexible pressure sensor arrays onto our bodies to monitor our health and help us pursue comfortable lives. A flexible sensor can collect signals from the human body and transfer the data to a computer, so this technology can be used for diagnosis, observation, and sharing [59].

Pedobarography can be used for the biomechanical analysis of gait recognition and posture [113]. Flexible pressure sensor arrays can detect the pressure between the plantar surface of a foot and the floor. This is meaningful for wearable biosensors, sports injury detection, and early diagnosis.

**Figure 9:**
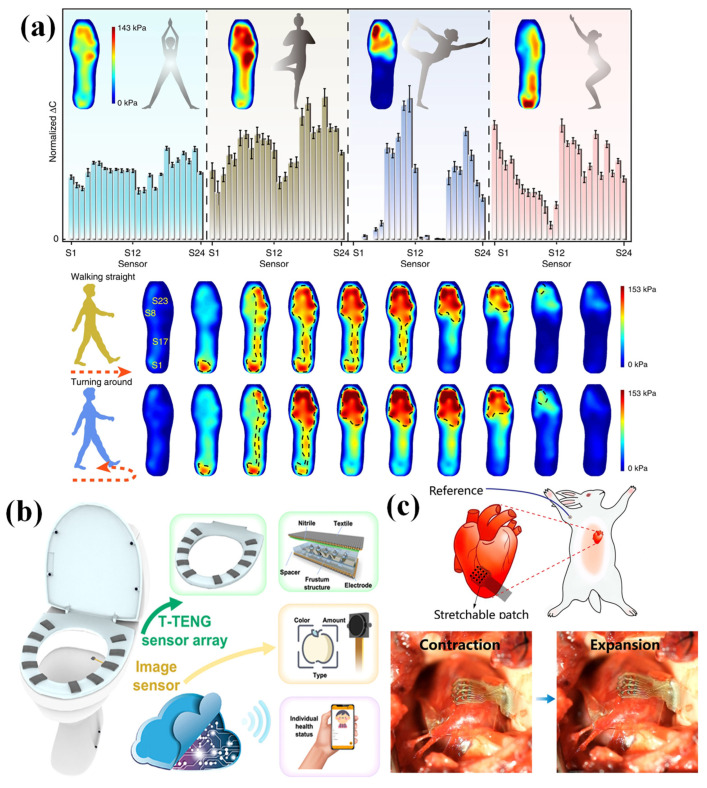
(**a**) Real-time pressure mapping in a smart insole system based on a sensor array. Reproduced with permission from Ref. [115]. Copyright 2020, Juan Tao et al. (**b**) Artificial intelligence toilet (AI-Toilet) for an integrated health monitoring system using smart triboelectric pressure sensor arrays. Reproduced with permission from Ref. [116]. Copyright 2021, Elsevier. (**c**) Intrinsically stretchable electronics with ultrahigh deformability to monitor dynamically moving organs. Reproduced with permission from Ref. [119]. Copyright 2022, Shaolei Wang et al.

By using PENGs serving as a sensor array, an insole plantar pressure mapping system was proposed [114]. This device could achieve self-powered operation, and the pressure signal was transferred to a smartphone for real-time foot pressure monitoring. An intelligent insole system that could realize static and dynamic plantar pressure mapping was proposed by Tao et al. [115]. This intelligent insole system is composed of capacitive pressure sensors and a data acquisition system with a wireless transmitter and a PC terminal. This device could detect static and dynamic pressure on the sole of the foot with different motions, such as different standing postures, yoga asana, walking straight (Figure 9a), etc. This device is beneficial in the restoration of healthy and normal posture.

As shown in Figure 9b, an intelligent toilet was demonstrated by using 10 textile-based triboelectric sensors to compose the toilet seat [116]. According to the triboelectric pressure sensor arrays, user identification and records of the entire seating time could be achieved, which is more private than the previous approach. The device can monitor a user’s health.

Implantable electronics are widely used in personalized health monitoring and precision therapies because of the development of biomedicine [117]. Flexible pressure sensor arrays can also be used in implantable electronics [118]. For example, Wang et al. fabricated a pressure sensor array to monitor dynamically moving organs [119]. In order to solve the problem of direct contact mapping of dynamic and fast-moving organs, a flexible pressure sensor array was attached to the surface of the right ventricle of a rabbit (Figure 9c). The electrophysiological mapping of the beating heart illustrated the potential of sensing arrays for implantable applications.

### 4.3. Aerospace

**Figure 10:**
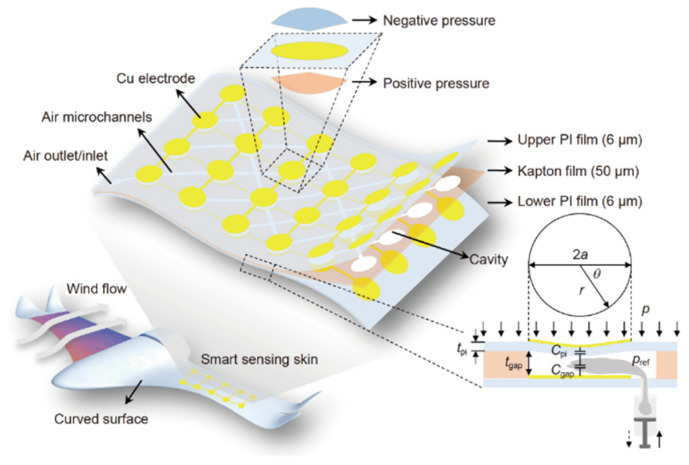
Conformable, programmable, and step-linear sensor array for large-range wind pressure measurements on a curved surface. Reproduced with permission from Ref. [122]. Copyright 2020, Science China Press and Springer-Verlag GmbH Germany, part of Springer Nature.

Morphing aircraft with intelligent variability and adaptability have become a research hotspot because they have a deformable structure to improve their aerodynamic performance [120]. So, it is crucial to accurately measure the parameter of such an aircraft’s structures and external environment. Recently, there have been more and more demands to apply flexible pressure sensor arrays in curved aircraft structures [121]. For example, to measure the pressure in wind-flow surroundings, a range-programmable capacitive pressure sensor array was demonstrated by Xiong et al. [122]. The sensing range of the capacitive array could be changed by changing the reference pressure. The capacitive sensor array was tested on an airfoil NACA0012 in a wind tunnel. It could detect the positive and negative stress, which was time-dynamic and space-distributed on curved surfaces (Figure 10).

## 5. Conclusions and Perspectives

At present, major achievements have been made in the research on flexible pressure sensors in order to enhance their performance. Table 1 summarizes the materials, sensitivity, and other performance parameters of the flexible pressure sensors discussed in this review.

Flexible pressure sensor arrays have received considerable attention due to their various applications. The development of micro-/nanoengineering and novel materials has resulted in significant advancements in flexible pressure sensor arrays. In this review, we introduce the recent advances in flexible pressure sensor arrays, including the working mechanisms, designs, advanced sensing performance, and device applications. The transmission mechanisms of flexible pressure sensor arrays primarily include piezoresistivity, capacitance, piezoelectricity, and triboelectricity, and their materials and structure design were summarized above. We sorted out the recent achievements of sensor arrays with different mechanisms and introduced the structures and the fabrication methods of these sensor arrays, etc. Flexible pressure sensors with high-density arrays can be fabricated by using micromachining technology to improve their resolution and sensitivity. Several methods have been introduced to improve the resolution and reduce crosstalk in pressure sensor arrays. Pressure sensor arrays based on the triboelectric and piezoelectric effects may reduce the power consumption or even have autonomous energy, which is very significant in the realization of the sustainable development of society. Flexible pressure sensor arrays might be widely used in various fields because they can achieve spatial pressure distribution monitoring and real-time trajectory mapping. Flexible pressure sensor arrays are also discussed extensively with respect to HMIs, healthcare, and the aerospace field.

In future research on flexible pressure sensor arrays, several factors require further consideration. First, flexible pressure sensors with high sensitivity over a wide pressure range are required for actual applications. The sensitivity and pressure-sensing range of sensors are contradictory due to the structural design of the sensors. In fact, as sensor signals can be amplified, increasing the sensing range is more important than increasing the sensitivity. Although various sensors can be selected according to the actual application requirements, it is important to investigate the pressure sensors with a wide sensing range. This requirement will be met by novel materials, structure designs, and transduction mechanisms. Second, flexible pressure sensor arrays are often deformed due to repeated deformation. Additional measures should be realized in order to improve the robustness and extend the life of flexible sensor arrays. Third, fabricating large-area and stable pressure sensor arrays is challenging. A large-scale integrated device platform with cost-effective fabrication methods should be investigated while considering the emerging 5G and Internet of Things technologies, which require massive sensor arrays. Fourth, the pressure sensors’ properties were tested in a laboratory, ignoring environmental factors such as temperature and humidity. Therefore, future research should focus on these implications for sensors. Fifth, sensing arrays’ performance should be improved further, such as with smaller pixel sizes and minimal crosstalk. Sixth, by combining sensor arrays with big data analysis, virtual space and real space will seamlessly interact in the future. Therefore, future research on flexible pressure sensor arrays and smart systems is expected to coincide with advances in signal processing and computation. We believe that more interdisciplinary collaborations are needed to advance this emerging field given the challenges and changes in flexible pressure sensor arrays.

## Figures and Tables

**Table 1:** Comparative table detailing the four types of sensors discussed.

Transduction Principles	Materials	Working Range	Sensitivity	Response Time	Stability	Ref.
Piezoresistivity	Multiwalled carbon nanotubes	0–21 kPa	1.10 kPa^−1^	29 ms	-	[60]
	Multiwalled carbon nanotubes	-	2.21 N^−1^	10/18 ms	-	[61]
	rGO@PolyHIPE foam	0–200 kPa	2.53 kPa^−1^ (0–140 kPa), 0.21 kPa^−^^1^ (140–120 kPa) 0.06 kPa^−^^1^ (120–200 kPa)	15.4 ms	10,000	[63]
	Organic thermoelectric materials	0.1–20 kPa	28.9 kPa^−1^	20 ms	10,000	[64]
	Urchin-like hollow carbon spheres	1–10,000 Pa	260.3 kPa^−1^	30/60 ms	5000	[65]
Capacitance	Air gap	40 kPa	14.268 kPa^−1^	50 ms	10,000	[71]
	Conductive fiber	0–20 kPa	0.21 kPa^−1^	10 ms	10,000	[74]
	Air gap	0–30 kPa	6.55% kPa^−^^1^ (0–16 kPa) 1.15% kPa^−^^1^ (16–30 kPa)	70 ms	2500	[75]
	PVA/H_3_PO_4_	0.08 Pa–360 kPa	220 kPa^−^^1^	9/18 ms	5000	[76]
Piezoelectricity	PVDF	-	1.2 V/N	-	-	[79]
	P(VDF-TrFE) micropillar	-	228.2 mV N^−1^	-	-	[80]
	PVDF	-	7.7 mV kPa^−1^	10 ms	80,000	[81]
	PEDOT:PSS and ZnO NWs	40–100 MPa	-	-	300	[83]
	ZnS:Mn particles	0.6–50 MPa	2.2 cps kPa^−1^	10 ms	10,000	[86]
Triboelectricity	PTFE film	0.1–37.5 kPa	53.7 mV Pa^−1^ (0.1–0.7 kPa) 5.3 mV Pa^−1^ (2.5–21.5 kPa) 1.2 mV Pa^−1^ (25.5–37.5 kPa)	-	30,000	[94]
	PDMS	1–80 kPa	0.06 kPa^−1^	-	10,000	[91]
	Ecoflex and PVA/PEI	5–50 kPa	0.063 V kPa^−1^	-	2250	[92]
	PDMS and metallized nanofibers	-	-	70 ms	12,000	[95]
	PDMS and carbon fiber	0.8–28.2 kPa	0.055 nA K Pa^−1^	68 ms	-	[97]

## Data Availability

Not applicable.

## References

[1] Mishra R.B., El-Atab N., Hussain A.M., Hussain M.M. (2021). Recent progress on flexible capacitive pressure sensors: From design and materials to applications. Adv. Mater. Technol..

[2] Wang L., Wang Z., Li Y., Luo Y., Lu B., Gao Y., Yu W., Gao G., Ding S. (2022). An ionically conductive, self-powered and stable organogel for pressure sensing. Nanomaterials.

[3] Zhai L., Gao L., Wang Z., Dai K., Wu S., Mu X. (2022). An energy harvester coupled with a triboelectric mechanism and electrostatic mechanism for biomechanical energy harvesting. Nanomaterials.

[4] Huang Y., Wan L., Jiang J., Li L., Zhai J. (2022). Self-powered resistance-switching properties of Pr0.7Ca0.3MnO3 film driven by triboelectric nanogenerator. Nanomaterials.

[5] Dong J., Wang C., Fan X., Wei L., Shen G., Sun R., Li R. (2022). Mechanical and dielectric properties of a flexible anisotropic rubber-based composite. Nanomaterials.

[6] Park C., Choi M., Lee S., Kim H., Lee T., Billah M.M., Jung B., Jang J. (2022). Highly sensitive, stretchable pressure sensor using blue laser annealed CNTs. Nanomaterials.

[7] Yang J.C., Mun J., Kwon S.Y., Park S., Bao Z., Park S. (2019). Electronic skin: Recent progress and future prospects for skin-attachable devices for health monitoring, robotics, and prosthetics. Adv. Mater..

[8] Zhong R.Y., Xu X., Klotz E., Newman S.T. (2017). Intelligent manufacturing in the context of industry 4.0: A review. Engineering.

[9] Zhang Z., Wen F., Sun Z., Guo X., He T., Lee C. (2022). Artificial intelligence-enabled sensing technologies in the 5G/internet of things era: From virtual reality/augmented reality to the digital twin. Adv. Intell. Syst..

[10] Su Y., Ma K., Mao X., Liu M., Zhang X. (2022). Highly compressible and sensitive flexible piezoresistive pressure sensor based on MWCNTs/Ti3C2Tx MXene @ melamine foam for human gesture monitoring and recognition. Nanomaterials.

[11] Pyo S., Lee J., Bae K., Sim S., Kim J. (2021). Recent progress in flexible tactile sensors for human-interactive systems: From sensors to advanced applications. Adv. Mater..

[12] Wang C., Xia K., Wang H., Liang X., Yin Z., Zhang Y. (2019). Advanced carbon for flexible and wearable electronics. Adv. Mater..

[13] Kim S., Amjadi M., Lee T.-I., Jeong Y., Kwon D., Kim M.S., Kim K., Kim T.S., Oh Y.S., Park I. (2019). Wearable, ultrawide-range, and bending-insensitive pressure sensor based on carbon nanotube network-coated porous elastomer sponges for human interface and healthcare devices. ACS Appl. Mater. Interfaces.

[14] Ai Y., Hsu T.H., Wu D.C., Lee L., Chen J.-H., Chen Y.-Z., Wu S.-C., Wu C., Wang Z.M., Chueh Y.-L. (2018). An ultrasensitive flexible pressure sensor for multimodal wearable electronic skins based on large-scale polystyrene ball@reduced graphene-oxide core–shell nanoparticles. J. Mater. Chem. C.

[15] Lu Y., Tian M., Sun X., Pan N., Chen F., Zhu S., Zhang X., Chen S. (2019). Highly sensitive wearable 3D piezoresistive pressure sensors based on graphene coated isotropic non-woven substrate. Compos. Part A-Appl. Sci..

[16] Zhang Y., Wang L., Zhao L., Wang K., Zheng Y., Yuan Z., Wang D., Fu X., Shen G., Han W. (2021). Flexible self-powered integrated sensing system with 3D periodic ordered black phosphorus@MXene thin-films. Adv. Mater..

[17] Wang J.-C., Karmakar R.S., Lu Y.-J., Huang C.-Y., Wei K.-C. (2015). Characterization of piezoresistive PEDOT:PSS pressure sensors with inter-digitated and cross-point electrode structures. Sensors.

[18] Lv B., Chen X., Liu C. (2020). A highly sensitive piezoresistive pressure sensor based on graphene Oxide/Polypyrrole@Polyurethane sponge. Sensors.

[19] Park J., Kim J., Hong J., Lee H., Lee Y., Cho S., Kim S.-W., Kim J.J., Kim S.Y., Ko H. (2018). Tailoring force sensitivity and selectivity by microstructure engineering of multidirectional electronic skins. NPG Asia Mater..

[20] Park J., Lee Y., Hong J., Ha M., Jung Y.-D., Lim H., Kim S.Y., Ko H. (2014). Giant tunneling piezoresistance of composite elastomers with interlocked microdome arrays for ultrasensitive and multimodal electronic skins. ACS Nano.

[21] Zhu B., Niu Z., Wang H., Leow W.R., Wang H., Li Y., Zheng L., Wei J., Huo F., Chen X. (2014). Microstructured graphene arrays for highly sensitive flexible tactile sensors. Small.

[22] Jeong C., Ko H., Kim H.-T., Sun K., Kwon T.-H., Jeong H.E., Park Y.-B. (2020). Bioinspired, high-sensitivity mechanical sensors realized with hexagonal microcolumnar arrays coated with ultrasonic-sprayed single-walled carbon nanotubes. ACS Appl. Mater. Interfaces.

[23] Ha M., Lim S., Park J., Um D.-S., Lee Y., Ko H. (2015). Bioinspired interlocked and hierarchical design of ZnO nanowire arrays for static and dynamic pressure-sensitive electronic skins. Adv. Funct. Mater..

[24] Pan L., Chortos A., Yu G., Wang Y., Isaacson S., Allen R., Shi Y., Dauskardt R., Bao Z. (2014). An ultra-sensitive resistive pressure sensor based on hollow-sphere microstructure induced elasticity in conducting polymer film. Nat. Commun..

[25] Zhang K., Ma Z., Deng H., Fu Q. (2022). Improving high-temperature energy storage performance of PI dielectric capacitor films through boron nitride interlayer. Adv. Compos. Hybrid Mater..

[26] Su M., Li P., Liu X., Wei D., Yang J. (2022). Textile-based flexible capacitive pressure sensors: A review. Nanomaterials.

[27] Nie B., Li R., Cao J., Brandt J.D., Pan T. (2015). Flexible transparent iontronic film for interfacial capacitive pressure sensing. Adv. Mater..

[28] Lipomi D.J., Vosgueritchian M., Tee B.C., Hellstrom S.L., Lee J.A., Fox C.H., Bao Z. (2011). Skin-like pressure and strain sensors based on transparent elastic films of carbon nanotubes. Nat. Nanotechnol..

[29] Wang J., Jiu J., Nogi M., Sugahara T., Nagao S., Koga H., He P., Suganuma K. (2015). A highly sensitive and flexible pressure sensor with electrodes and elastomeric interlayer containing silver nanowires. Nanoscale.

[30] Ho D.H., Sun Q., Kim S.Y., Han J.T., Kim D.H., Cho J.H. (2016). Stretchable and multimodal all graphene electronic skin. Adv. Mater..

[31] Yang J.C., Kim J.-O., Oh J., Kwon S.Y., Sim J.Y., Kim D.W., Choi H.B., Park S. (2019). Microstructured porous pyramid-based ultrahigh sensitive pressure sensor insensitive to strain and temperature. ACS Appl. Mater. Interfaces.

[32] Chhetry A., Sharma S., Yoon H., Ko S., Park J.Y. (2020). Enhanced sensitivity of capacitive pressure and strain sensor based on CaCu_3_Ti_4_O_12_ wrapped hybrid sponge for wearable applications. Adv. Funct. Mater..

[33] Choi J., Kwon D., Kim K., Park J., Orbe D.D., Gu J., Ahn J., Cho I., Jeong Y., Oh Y. (2020). Synergetic effect of porous elastomer and percolation of carbon nanotube filler toward high performance capacitive pressure sensors. ACS Appl. Mater. Interfaces.

[34] Mannsfeld S.C.B., Tee B.C.-K., Stoltenberg R.M., Chen C.V.H.-H., Barman S., Muir B.V.O., Sokolov A.N., Reese C., Bao Z. (2010). Highly sensitive flexible pressure sensors with microstructured rubber dielectric layers. Nat. Mater..

[35] Guo Y., Gao S., Yue W., Zhang C., Li Y. (2019). Anodized aluminum oxide-assisted low-cost flexible capacitive pressure sensors based on double-sided nanopillars by a facile fabrication method. ACS Appl. Mater. Interfaces.

[36] Miller S., Bao Z. (2015). Fabrication of flexible pressure sensors with microstructured polydimethylsiloxane dielectrics using the breath figures method. J. Mater. Res..

[37] Hsieh G.W., Shih L.C., Chen P.Y. (2022). Porous polydimethylsiloxane elastomer hybrid with zinc oxide nanowire for wearable, wide-range, and low detection limit capacitive pressure sensor. Nanomaterials.

[38] Hwang J., Kim Y., Yang H., Oh J.H. (2021). Fabrication of hierarchically porous structured PDMS composites and their application as a flexible capacitive pressure sensor. Compos. Part B Eng..

[39] Atalay O., Atalay A., Gafford J., Walsh C. (2017). A highly sensitive capacitive-based soft pressure sensor based on a conductive fabric and a microporous dielectric layer. Adv. Mater. Technol..

[40] Dai K., Wang X., Niu S., Yi F., Yin Y., Chen L., Zhang Y., You Z. (2016). Simulation and structure optimization of triboelectric nanogenerators considering the effects of parasitic capacitance. Nano Res..

[41] Mu J., Xian S., Yu J., Zhao J., Song J., Li Z., Hou X., Chou X., He J. (2022). Synergistic enhancement properties of a flexible integrated pan/pvdf piezoelectric sensor for human posture recognition. Nanomaterials.

[42] Wang Z.L., Song J. (2006). Piezoelectric nanogenerators based on zinc oxide nanowire arrays. Science.

[43] Ma M., Zhang Z., Zhao Z., Liao Q., Kang Z., Gao F., Zhao X., Zhang Y. (2019). Self-powered flexible antibacterial tactile sensor based on triboelectric-piezoelectric-pyroelectric multi-effect coupling mechanism. Nano Energy.

[44] Kim K.-B., Jang W., Cho J.Y., Woo S.B., Jeon D.H., Ahn J.H., Hong S.D., Koo H.Y., Sung T.H. (2018). Transparent and flexible piezoelectric sensor for detecting human movement with a boron nitride nanosheet (BNNS). Nano Energy.

[45] Kim M.-O., Pyo S., Oh Y., Kang Y., Cho K.-H., Choi J., Kim J. (2018). Flexible and multi-directional piezoelectric energy harvester for self-powered human motion sensor. Smart Mater. Struct..

[46] Pi Z., Zhang J., Wen C., Zhang Z.-b., Wu D. (2014). Flexible piezoelectric nanogenerator made of poly(vinylidenefluoride-co-trifluoroethylene) (PVDF-TrFE) thin film. Nano Energy.

[47] Fan F.-R., Tian Z.-Q., Lin Wang Z. (2012). Flexible triboelectric generator. Nano Energy.

[48] Wang Z.L., Wang A.C. (2019). On the origin of contact-electrification. Mater. Today.

[49] Park S., Kim H., Vosgueritchian M., Cheon S., Kim H., Koo J.H., Kim T.R., Lee S., Schwartz G., Chang H. (2014). Stretchable energy-harvesting tactile electronic skin capable of differentiating multiple mechanical stimuli modes. Adv. Mater..

[50] Zhou K., Zhao Y., Sun X., Yuan Z., Zheng G., Dai K., Mi L., Pan C., Liu C., Shen C. (2020). Ultra-stretchable triboelectric nanogenerator as high-sensitive and self-powered electronic skins for energy harvesting and tactile sensing. Nano Energy.

[51] He F., You X., Gong H., Yang Y., Bai T., Wang W., Guo W., Liu X., Ye M. (2020). Stretchable, biocompatible, and multifunctional silk fibroin-based hydrogels toward wearable strain/pressure sensors and triboelectric nanogenerators. ACS Appl. Mater. Interfaces.

[52] Huang J., Yang X., Yu J., Han J., Jia C., Ding M., Sun J., Cao X., Sun Q., Wang Z.L. (2020). A universal and arbitrary tactile interactive system based on self-powered optical communication. Nano Energy.

[53] Pyo S., Kim M.-O., Kwon D.-S., Kim W., Yang J.-H., Cho H.S., Lee J.H., Kim J. (2020). All-textile wearable triboelectric nanogenerator using pile-embroidered fibers for enhancing output power. Smart Mater. Struct..

[54] Li H., Fang X., Li R., Liu B., Tang H., Ding X., Xie Y., Zhou R., Zhou G., Tang Y. (2020). All-printed soft triboelectric nanogenerator for energy harvesting and tactile sensing. Nano Energy.

[55] Luo Y., Li Y., Feng X., Pei Y., Zhang Z., Wang L., Zhao Y., Lu B., Zhu B. (2020). Triboelectric nanogenerators with porous and hierarchically structured silk fibroin films via water electrospray-etching technology. Nano Energy.

[56] Maharjan P., Bhatta T., Salauddin M., Rasel M.S., Rahman M.T., Rana S.M.S., Park J.Y. (2020). A human skin-inspired self-powered flex sensor with thermally embossed microstructured triboelectric layers for sign language interpretation. Nano Energy.

[57] Zhou Z., Weng L., Tat T., Libanori A., Lin Z., Ge L., Yang J., Chen J. (2020). Smart insole for robust wearable biomechanical energy harvesting in harsh environments. ACS Nano.

[58] Ha M., Lim S., Cho S., Lee Y., Na S., Baig C., Ko H. (2018). Skin-inspired hierarchical polymer architectures with gradient stiffness for spacer-free, ultrathin, and highly sensitive triboelectric sensors. ACS Nano.

[59] Amjadi M., Kyung K.-U., Park I., Sitti M. (2016). Stretchable, skin-mountable, and wearable strain sensors and their potential applications: A review. Adv. Funct. Mater..

[60] Wang L., Peng H., Wang X., Chen X., Yang C., Yang B., Liu J. (2016). PDMS/MWCNT-based tactile sensor array with coplanar electrodes for crosstalk suppression. Microsyst. Nanoeng..

[61] Park J., Lee Y., Hong J., Lee Y., Ha M., Jung Y., Lim H., Kim S.Y., Ko H. (2014). Tactile-direction-sensitive and stretchable electronic skins based on human-skin-inspired interlocked microstructures. ACS Nano.

[62] Lee Y., Park J., Cho S., Shin Y.-E., Lee H., Kim J., Myoung J., Cho S., Kang S., Baig C. (2018). Flexible ferroelectric sensors with ultrahigh pressure sensitivity and linear response over exceptionally broad pressure range. ACS Nano.

[63] Yang L., Liu Y., Filipe C.D.M., Ljubic D., Luo Y., Zhu H., Yan J., Zhu S. (2019). Development of a highly sensitive, broad-range hierarchically structured reduced graphene oxide/polyhipe foam for pressure sensing. ACS Appl. Mater. Interfaces.

[64] Zhang F., Zang Y., Huang D., Di C.-A., Zhu D. (2015). Flexible and self-powered temperature-pressure dual-parameter sensors using microstructure-frame-supported organic thermoelectric materials. Nat. Commun..

[65] Shi L., Li Z., Chen M., Qin Y., Jiang Y., Wu L. (2020). Quantum effect-based flexible and transparent pressure sensors with ultrahigh sensitivity and sensing density. Nat. Commun..

[66] Lee J.H., Heo J.S., Kim Y.-J., Eom J., Jung H.J., Kim J.-W., Kim I., Park H.-H., Mo H.S., Kim Y.-H. (2020). A behavior-learned cross-reactive sensor matrix for intelligent skin perception. Adv. Mater..

[67] Sundaram S., Kellnhofer P., Li Y., Zhu J.-Y., Torralba A., Matusik W. (2019). Learning the signatures of the human grasp using a scalable tactile glove. Nature.

[68] Ge J., Sun L., Zhang F.-R., Zhang Y., Shi L.-A., Zhao H.-Y., Zhu H.-W., Jiang H.-L., Yu S.-H. (2016). A stretchable electronic fabric artificial skin with pressure-, lateral strain-, and flexion-sensitive properties. Adv. Mater..

[69] Wang Y., Zhu J., Shen M., Gao C., Wang P., Zhou W., Zhao C., Muhammad N., Gao J., Gao Q. (2022). Three-layer core-shell Ag/AgCl/PEDOT: PSS composite fibers via a one-step single-nozzle technique enabled skin-inspired tactile sensors. Chem. Eng. J..

[70] Yang W., Li N.-W., Zhao S., Yuan Z., Wang J., Du X., Wang B., Cao R., Li X., Xu W. (2018). A breathable and screen-printed pressure sensor based on nanofiber membranes for electronic skins. Adv. Mater. Technol..

[71] Zeng X., Wang Z., Zhang H., Yang W., Xiang L., Zhao Z., Peng L.-M., Hu Y. (2019). Tunable, ultrasensitive, and flexible pressure sensors based on wrinkled microstructures for electronic skins. ACS Appl. Mater. Interfaces.

[72] Zhao X., Hua Q., Yu R., Zhang Y., Pan C. (2015). Flexible, stretchable and wearable multifunctional sensor array as artificial electronic skin for static and dynamic strain mapping. Adv. Electron. Mater..

[73] Hua Q., Sun J., Liu H., Bao R., Yu R., Zhai J., Pan C., Wang Z.L. (2018). Skin-inspired highly stretchable and conformable matrix networks for multifunctional sensing. Nat. Commun..

[74] Lee J., Kwon H., Seo J., Shin S., Koo J.H., Pang C., Son S., Kim J.H., Jang Y.H., Kim D.E. (2015). Conductive fiber-based ultrasensitive textile pressure sensor for wearable electronics. Adv. Mater..

[75] Pyo S., Choi J., Kim J. (2018). Flexible, transparent, sensitive, and crosstalk-free capacitive tactile sensor array based on graphene electrodes and air dielectric. Adv. Electron. Mater..

[76] Bai N., Wang L., Wang Q., Deng J., Wang Y., Lu P., Huang J., Li G., Zhang Y., Yang J. (2020). Graded intrafillable architecture-based iontronic pressure sensor with ultra-broad-range high sensitivity. Nat. Commun..

[77] Wang X., Song W.-Z., You M.-H., Zhang J., Yu M., Fan Z., Ramakrishna S., Long Y.-Z. (2018). Bionic single-electrode electronic skin unit based on piezoelectric nanogenerator. ACS Nano.

[78] Zirkl M., Scheipl G., Stadlober B., Rendl C., Greindl P. PyzoFlex: A printed piezoelectric pressure sensing foil for human machine interfaces. Proceedings of the Organic Field-Effect Transistors XII; and Organic Semiconductors in Sensors and Bioelectronics VI.

[79] Emamian S., Narakathu B.B., Chlaihawi A.A., Bazuin B.J., Atashbar M.Z. (2017). Screen printing of flexible piezoelectric based device on polyethylene terephthalate (PET) and paper for touch and force sensing applications. Sens. Actuators A.

[80] Chen X., Shao J., Tian H., Li X., Wang C., Luo Y., Li S. (2020). Scalable imprinting of flexible multiplexed sensor arrays with distributed piezoelectricity-enhanced micropillars for dynamic tactile sensing. Adv. Mater. Technol..

[81] Lin W., Wang B., Peng G., Shan Y., Hu H., Yang Z. (2021). Skin-inspired piezoelectric tactile sensor array with crosstalk-free row + column electrodes for spatiotemporally distinguishing diverse stimuli. Adv. Sci..

[82] Pan C., Dong L., Zhu G., Niu S., Yu R., Yang Q., Liu Y., Wang Z.L. (2013). High-resolution electroluminescent imaging of pressure distribution using a piezoelectric nanowire LED array. Nat. Photonics.

[83] Bao R., Wang C., Dong L., Yu R., Zhao K., Wang Z.L., Pan C. (2015). Flexible and controllable piezo-phototronic pressure mapping sensor matrix by ZnO NW/p-Polymer LED array. Adv. Funct. Mater..

[84] Peng Y., Que M., Lee H.E., Bao R., Wang X., Lu J., Yuan Z., Li X., Tao J., Sun J. (2019). Achieving high-resolution pressure mapping via flexible GaN/ZnO nanowire LEDs array by piezo-phototronic effect. Nano Energy.

[85] Bao R., Wang C., Dong L., Shen C., Zhao K., Pan C. (2016). CdS nanorods/organic hybrid LED array and the piezo-phototronic effect of the device for pressure mapping. Nanoscale.

[86] Wang X., Zhang H., Yu R., Dong L., Peng D., Zhang A., Zhang Y., Liu H., Pan C., Wang Z.L. (2015). Dynamic pressure mapping of personalized handwriting by a flexible sensor matrix based on the mechanoluminescence process. Adv. Mater..

[87] Zhou Y., Shen M., Cui X., Shao Y., Li L., Zhang Y. (2021). Triboelectric nanogenerator based self-powered sensor for artificial intelligence. Nano Energy.

[88] Lin L., Xie Y., Wang S., Wu W., Niu S., Wen X., Wang Z.L. (2013). Triboelectric active sensor array for self-powered static and dynamic pressure detection and tactile imaging. ACS Nano.

[89] Wang X., Dong L., Zhang H., Yu R., Pan C., Wang Z.L. (2015). Recent progress in electronic skin. Adv. Sci..

[90] Yang Y., Zhang H., Lin Z.-H., Zhou Y.S., Jing Q., Su Y., Yang J., Chen J., Hu C., Wang Z.L. (2013). Human skin based triboelectric nanogenerators for harvesting biomechanical energy and as self-powered active tactile sensor system. ACS Nano.

[91] Wang X., Zhang H., Dong L., Han X., Du W., Zhai J., Pan C., Wang Z.L. (2016). Self-powered high-resolution and pressure-sensitive triboelectric sensor matrix for real-time tactile mapping. Adv. Mater..

[92] Wang L., Liu Y., Liu Q., Zhu Y., Wang H., Xie Z., Yu X., Zi Y. (2020). A metal-electrode-free, fully integrated, soft triboelectric sensor array for self-powered tactile sensing. Microsyst. Nanoeng..

[93] Jiang X.-Z., Sun Y.-J., Fan Z., Zhang T.-Y. (2016). Integrated flexible, waterproof, transparent, and self-powered tactile sensing panel. ACS Nano.

[94] Wang H.L., Kuang S.Y., Li H.Y., Wang Z.L., Zhu G. (2020). Large-area integrated triboelectric sensor array for wireless static and dynamic pressure detection and mapping. Small.

[95] Wang X., Zhang Y., Zhang X., Huo Z., Li X., Que M., Peng Z., Wang H., Pan C. (2018). A highly stretchable transparent self-powered triboelectric tactile sensor with metallized nanofibers for wearable electronics. Adv. Mater..

[96] Luo Y., Xiao X., Chen J., Li Q., Fu H. (2022). Machine-learning-assisted recognition on bioinspired soft sensor arrays. ACS Nano.

[97] Ma M., Zhang Z., Liao Q., Yi F., Han L., Zhang G., Liu S., Liao X., Zhang Y. (2017). Self-powered artificial electronic skin for high-resolution pressure sensing. Nano Energy.

[98] Zhu X.X., Meng X.S., Kuang S.Y., Wang X.D., Pan C.F., Zhu G., Wang Z.L. (2017). Triboelectrification-enabled touch sensing for self-powered position mapping and dynamic tracking by a flexible and area-scalable sensor array. Nano Energy.

[99] Wang X., Que M., Chen M., Han X., Li X., Pan C., Wang Z.L. (2017). Full dynamic-range pressure sensor matrix based on optical and electrical dual-mode sensing. Adv. Mater..

[100] Wang S., Xu J., Wang W., Wang G.-J.N., Rastak R., Molina-Lopez F., Chung J.W., Niu S., Feig V.R., Lopez J. (2018). Skin electronics from scalable fabrication of an intrinsically stretchable transistor array. Nature.

[101] Yang Z.W., Pang Y., Zhang L., Lu C., Chen J., Zhou T., Zhang C., Wang Z.L. (2016). Tribotronic transistor array as an active tactile sensing system. ACS Nano.

[102] Gao G., Wan B., Liu X., Sun Q., Yang X., Wang L., Pan C., Wang Z.L. (2018). Tunable tribotronic dual-gate logic devices based on 2D MoS_2_ and black phosphorus. Adv. Mater..

[103] Liu Y., Yang W., Yan Y., Wu X., Wang X., Zhou Y., Hu Y., Chen H., Guo T. (2020). Self-powered high-sensitivity sensory memory actuated by triboelectric sensory receptor for real-time neuromorphic computing. Nano Energy.

[104] Wei X.Y., Wang X., Kuang S.Y., Su L., Li H.Y., Wang Y., Pan C., Wang Z.L., Zhu G. (2016). Dynamic triboelectrification-induced electroluminescence and its use in visualized sensing. Adv. Mater..

[105] Wang Y., Wang H.L., Li H.Y., Wei X.Y., Wang Z.L., Zhu G. (2019). Enhanced high-resolution triboelectrification-induced electroluminescence for self-powered visualized interactive sensing. ACS Appl. Mater. Interfaces.

[106] Wei X.Y., Liu L., Wang H.L., Kuang S.Y., Zhu X., Wang Z.L., Zhang Y., Zhu G. (2018). High-intensity triboelectrification-induced electroluminescence by microsized contacts for self-powered display and illumination. Adv. Mater. Interfaces.

[107] Jia C., Xia Y., Zhu Y., Wu M., Zhu S., Wang X. (2022). High-brightness, high-resolution, and flexible triboelectrification-induced electroluminescence skin for real-time imaging and human-machine information interaction. Adv. Funct. Mater..

[108] Bouton C.E., Shaikhouni A., Annetta N.V., Bockbrader M.A., Friedenberg D.A., Nielson D.M., Sharma G., Sederberg P.B., Glenn B.C., Mysiw W.J. (2016). Restoring cortical control of functional movement in a human with quadriplegia. Nature.

[109] Luo S., Bimbo J., Dahiya R., Liu H. (2017). Robotic tactile perception of object properties: A review. Mechatronics.

[110] Xu J., Li X., Chang H., Zhao B., Tan X., Yang Y., Tian H., Zhang S., Ren T.L. (2022). Electrooculography and tactile perception collaborative interface for 3D human-machine interaction. ACS Nano.

[111] Niu H., Gao S., Yue W., Li Y., Zhou W., Liu H. (2020). Highly morphology-controllable and highly sensitive capacitive tactile sensor based on epidermis-dermis-inspired interlocked asymmetric-nanocone arrays for detection of tiny pressure. Small.

[112] Ozioko O., Dahiya R. (2021). Smart tactile gloves for haptic interaction, communication, and rehabilitation. Adv. Intell. Syst..

[113] Zulkifli S.S., Loh W.P. (2020). A state-of-the-art review of foot pressure. Foot Ankle Surg..

[114] Deng C., Tang W., Liu L., Chen B., Li M., Wang Z.L. (2018). Self-powered insole plantar pressure mapping system. Adv. Funct. Mater..

[115] Tao J., Dong M., Li L., Wang C., Li J., Liu Y., Bao R., Pan C. (2020). Real-time pressure mapping smart insole system based on a controllable vertical pore dielectric layer. Microsyst. Nanoeng..

[116] Zhang Z., Shi Q., He T., Guo X., Dong B., Lee J., Lee C. (2021). Artificial intelligence of toilet (AI-Toilet) for an integrated health monitoring system (IHMS) using smart triboelectric pressure sensors and image sensor. Nano Energy.

[117] Song Y., Min J., Gao W. (2019). Wearable and implantable electronics: Moving toward precision therapy. ACS Nano.

[118] Wang C., Shi Q., Lee C. (2022). Advanced implantable biomedical devices enabled by triboelectric nanogenerators. Nanomaterials.

[119] Wang S., Nie Y., Zhu H., Xu Y., Cao S., Zhang J., Li Y., Wang J., Ning X., Kong D. (2022). Intrinsically stretchable electronics with ultrahigh deformability to monitor dynamically moving organs. Sci. Adv..

[120] Sun J., Guan Q., Liu Y., Leng J. (2016). Morphing aircraft based on smart materials and structures: A state-of-the-art review. J. Intell. Mater. Syst. Struct..

[121] Huang Y., Zhu C., Xiong W., Wang Y., Jiang Y., Qiu L., Guo D., Hou C., Jiang S., Yang Z. (2022). Flexible smart sensing skin for “Fly-by-Feel” morphing aircraft. Sci. China Technol. Sci..

[122] Xiong W., Guo D., Yang Z., Zhu C., Huang Y. (2020). Conformable, programmable and step-linear sensor array for large-range wind pressure measurement on curved surface. Sci. China Technol. Sci..

